# Overcoming pH defenses on the skin to establish infections

**DOI:** 10.1371/journal.ppat.1010512

**Published:** 2022-05-26

**Authors:** Flavia G. Costa, Alexander R. Horswill

**Affiliations:** 1 Department of Immunology and Microbiology, University of Colorado School of Medicine, Aurora, Colorado, United States of America; 2 Department of Veterans Affairs, Eastern Colorado Healthcare System, Aurora, Colorado, United States of America; Nanyang Technological University, SINGAPORE

## Abstract

Skin health is influenced by the composition and integrity of the skin barrier. The healthy skin surface is an acidic, hypertonic, proteinaceous, and lipid-rich environment that microorganisms must adapt to for survival, and disruption of this environment can result in dysbiosis and increase risk for infectious diseases. This work provides a brief overview of skin barrier function and skin surface composition from the perspective of how the most common skin pathogen, *Staphylococcus aureus*, combats acid stress. Advancements in replicating this environment in the laboratory setting for the study of *S*. *aureus* pathogenesis on the skin, as well as future directions in this field, are also discussed.

## The skin surface environment

**Fig 1:**
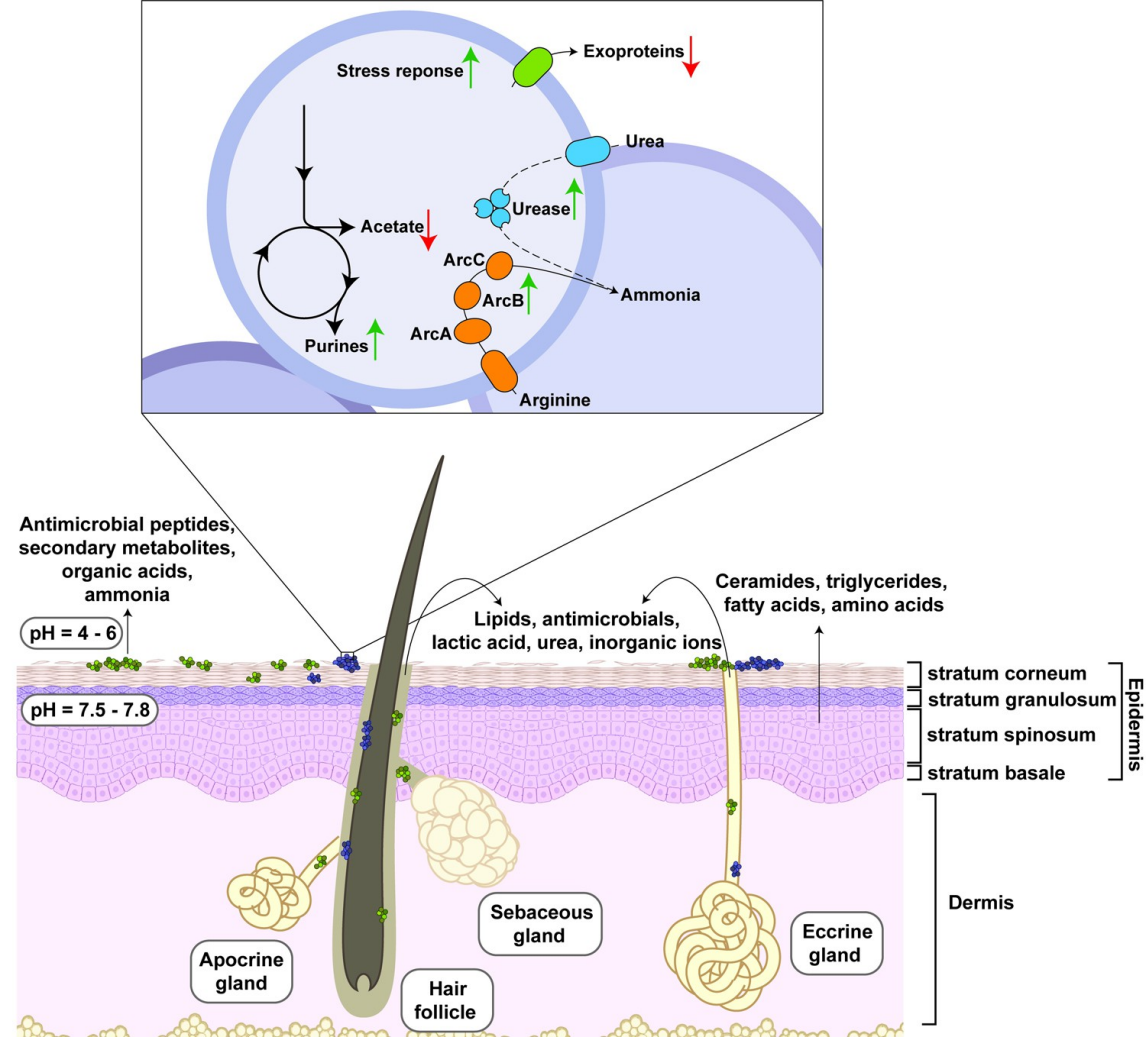
*Staphylococcus aureus* response to the skin barrier environment. The skin, composed of the epidermis and dermis, form a physical and chemical barrier against microbial pathogens. Additionally, commensals (depicted in green) produce secondary metabolites and antimicrobial peptides that further inhibit growth of opportunistic pathogens such as *S*. *aureus* (depicted in purple). The skin layers depicted in Fig 1 were made using BioRender. Inset: *S*. *aureus* responds to the acid stress caused by the skin surface environment by down-regulation of organic acid production and exotoxins and up-regulation of purine biosynthesis, stress response factors, and ammonia-producing pathways like the ACME-Arc and urease. The dotted line indicates that, although it is up-regulated, the role of urease in this environment has not been demonstrated. ACME, arginine catabolic mobile element.

The skin is the largest organ of the human body and forms a protective barrier against environmental hazards such as bacterial pathogens [1]. As depicted in **Fig 1**, skin is composed of 2 main layers: the outer epidermal layer, followed by the thicker dermal layer [1]. Keratinocytes are generated in the stratum basale of the epidermis and then differentiate as they move toward the skin surface. The outermost layer of mature keratinocytes, termed as “stratum corneum,” is the primary physical barrier of the skin surface and is rich in ceramides, triglycerides, amino acids, and free fatty acids [1].

In addition to the skin’s physical properties, the chemical environment of the skin surface presents a challenge to colonizing microorganisms [2]. This chemical composition is largely influenced by sweat and sebum. Sweat is an aqueous mixture composed of the excretions from eccrine and apocrine glands, whereas sebum is a hydrophobic substance made of secretions from the sebaceous glands. Eccrine glands are distributed throughout the skin and excrete inorganic ions (e.g., sodium, potassium, and chloride) and metabolic by-products such as lactic acid, ammonia, amino acids, and urea. Apocrine glands, which are primarily concentrated in the axilla and groin, excrete proteins, lipids, and steroids [3]. Sebaceous glands produce sebum, a viscous fluid composed of triglycerides, wax esters, squalene, and free fatty acids. Together, the stratum corneum and gland secretions produce a lipid-rich, saline, acidic environment enriched with organic acids, amino acids, and urea.

The acidic pH of human skin surface, often termed the “acid mantle,” is a key component of healthy skin. Recent investigations measuring skin surface pH in healthy volunteers report an average pH between 4.1 and 5.8 [4]. Organic and fatty acids excreted from the host as well as by-products of skin microbiota metabolic activity contribute to the acidity of the skin surface [5]. Additionally, activity of skin antimicrobial peptides, a key component of innate immunity, are optimal at acidic pH [6]. The application of cleansing agents as well as age, genetics, and inflammatory conditions can increase the skin pH and result in skin barrier disruption, dysbiosis, and disease (reviewed in [4]). Altogether, these observations indicate that skin pH likely represents a critical factor in maintaining a healthy skin environment and should be considered when characterizing microbial pathogenesis on the skin.

## Contribution of skin pH to *Staphylococcus aureus* pathogenesis

Acidic environments are associated with repression of virulence across several bacterial species [7]. Comparative transcriptional analyses of *S*. *aureus* grown in neutral and acidic media identified that several genetic factors involved in virulence and pathogenesis are repressed by acidic pH [8,9]. For example, transcription of several *S*. *aureus* exoprotein loci, including α-hemolysin (encoded by *hla*), aureolysin (*aur*), and toxic shock syndrome toxin 1 (*tst*), are repressed in acidic media [8]. This may be explained by repression of the regulator SaeS/R at low pH, a global regulator that controls expression of many exoproteins [8]. In a recently published study of *S*. *aureus* colonization of an ex vivo skin explant, both SaeS/R and the quorum sensing regulator Agr, which controls expression of phenol-soluble modulins and other virulence factors, were repressed in early stages of skin colonization [10]. This is in contrast to data collected from *S*. *aureus* abscesses in pediatric patients, where many genes in the SaeR/S regulon are up-regulated [11]. These data suggest that the environment of healthy human skin could prevent or delay *S*. *aureus* entering a virulent physiological state. Further investigation is needed to explore this connection between *S*. *aureus* virulence and the skin surface environment.

## Lactic acid stress elicits a shift in *S*. *aureus* metabolism and physiology

*S*. *aureus* growth is inhibited in media supplemented with lactic acid at a pH of 4.8, which suggests this acid present on the skin surface may disrupt *S*. *aureus* colonization [12]. Lactic acid is primarily excreted by the eccrine glands in concentrations ranging from 5 to 40 mM and is a major organic acid on the skin surface [13]. Lactic acid supplemented media induces several notable transcriptional changes affecting the metabolism of *S*. *aureus* [12], such as increased urease expression and rewiring central metabolic pathways away from acetate production. This metabolic adaptation is likely a concerted effort to increase intracellular pH via (1) production of ammonia; and (2) reduction of organic acid production [12,14]. Purine biosynthesis is also up-regulated during lactic acid stress or during challenge with other organic acid species [8,12]. Notably, hydrochloric acid results in inhibition of purine biosynthesis, demonstrating that the method of acidification may be important to consider when translating data collected *in vitro* to the host environment. Although the role of purine biosynthesis in the context of acid stress and skin colonization has not been studied, observations in other *S*. *aureus* infection models show that transcriptional increases in purine biosynthesis contribute to the stringent response and persistence [15]. In addition to metabolic changes, expression of several gene products with roles in mediating protein damage, such as chaperonins and Clp protease, are highly up-regulated during lactic acid stress. This may indicate a possible mechanism for lactic acid stress in damage of intracellular proteins, a role consistent with stress mechanisms by other organic acids [14]. Taken together, these data suggest that a lactic acid replete, acidic environment is metabolically and physiologically stressful for *S*. *aureus*, and this has necessitated the development of complex response mechanisms within *S*. *aureus* for counteracting lactic acid toxicity.

## Production of ammonia to combat acid stress

One approach to overcoming acid stress is by production of a basic metabolite, such as ammonia, which can be used to neutralize the toxic acid. Urease activity and the functions encoded in the arginine catabolic mobile element (ACME) have both been demonstrated in *S*. *aureus* strains to combat acid stress in this manner [16,17].

The genes encoding urease are highly up-regulated in response to low environmental pH [8,9,12]. Urease converts urea to ammonia and carbon dioxide via a Ni(II)-dependent reaction [18]. Urease activity is key to the pathogenesis of several microorganisms in several different organs of the body, including *Helicobacter pylori* in the stomach and *Staphylococcus saprophyticus* in the urinary tract [18]. *S*. *aureus* utilizes the ammonia produced from urea to neutralize the pH of acidic media, and this process contributes to its survival and pathogenesis in the murine kidney [16]. Although human sweat contains 4 to 12 mM urea, the role of urease activity on the human skin surface for *S*. *aureus* pH homeostasis has not been described [13].

The ACME is found in coagulase-negative Staphylococci and a subset of methicillin-resistant *S*. *aureus* strains, including most of the highly virulent USA300 type strains [19]. *S*. *aureus* has its own arginine deiminase (ADI) pathway that also converts arginine into L-ornithine, ammonia, and carbon dioxide; however, the independent transcriptional regulation of ACME allows for higher ADI activity in conditions of skin-like acidity [17]. Additionally, the ACME encodes a polyamine *N-*acetyl transferase (*speG*) that confers resistance to host polyamines produced in response to infection [17]. *S*. *aureus* is not the only skin pathogen that utilizes arginine deamination to increase the pH on the skin—this has also been shown with *Streptococcus pyogenes*, which uses its ADI pathway to raise pH on the skin and contribute to pathogenesis [20].

## Advances in modeling human skin infections

A significant bottleneck in researching microbe–host interactions on the skin is replicating the skin environment in a research setting [21]. The pH and metabolites present on human skin are unique to the species, which should also be considered when investigating topical skin infections with animal models [4]. Remarkable developments have been made in recent years in using human skin constructs for infection modeling in the laboratory, with several companies offering products and services using differentiated keratinocyte and fibroblast skin models and surgical *ex vivo* samples [21]. However, published work utilizing these models for *S*. *aureus* human skin colonization and pathogenesis do not recapitulate the acidity and metabolic composition of the skin surface, as *S*. *aureus* is often inoculated onto skin in a phosphate saline solution at a neutral pH, and the function of the secretion glands has not been confirmed in these models. Considering the transcriptional changes discussed earlier that respond to pH, these methodological decisions could affect *S*. *aureus* colonization and pathogenesis. To better understand how *S*. *aureus* interacts with the skin surface and acid mantle, researchers should consider incorporating these aspects into their model systems for *S*. *aureus* skin colonization and pathogenesis.

## Concluding remarks and future directions

During colonization and pathogenesis, *S*. *aureus* and other pathogens encounter acidic environments that impact metabolism and physiology, including the skin surface. In conditions mimicking the lactic acid replete, low pH skin environment, *S*. *aureus* combats acid-induced stress by acid neutralization with ammonia, reduction of organic acid production, and increased production of functions with possible damage repair mechanisms. Understanding microbe–host interactions using improved models for human skin colonization and infection will provide more insight into *S*. *aureus* physiology on the skin and provide opportunities for therapeutic intervention to lower risk for skin infections.
